# Sex and gender differences in cancer research and its application to clinical oncology and therapeutics

**DOI:** 10.1016/j.clinsp.2025.100670

**Published:** 2025-05-12

**Authors:** Rut Lucas-Domínguez, Cristina Rius, Yiming Liu, Andrea Sixto-Costoya, Juan Carlos Valderrama-Zurián

**Affiliations:** a UISYS Group, Department of History of Science and Information Science. Faculty of Medicine and Dentistry, University of Valencia, Spain; b Unit associated with the Interuniversity Institute for Advanced Research on the Evaluation of Science and the University (INAECU) UC3M-UAM, Spain; c CIBERONC, Valencia, Spain; d Spanish National Centre for Cardiovascular Research (CNIC), Madrid, Spain; e CIBERCV, Madrid, Spain; f Department of Social Work and Social Services, Faculty of Social Sciences, University of Valencia, Spain

**Keywords:** Cancer research, Women's health, Sex and gender differences, Gender bias, Gender gap

## Abstract

**Objectives:**

Oncology has exhibited gender inequalities in leadership and authorship, alongside significant differences in cancer incidence, mortality, and survival rates between sexes. These disparities highlight the urgent need for sex- and gender-specific scientific reporting to improve research accuracy and clinical applications and encourage the same opportunities in research careers.

**Methods:**

The integration of gender perspective in cancer was assessed by analyzing scientific publications comparing 2011 and 2021. The correlation between the gender of the authors and the different types of cancer studied was examined, as well as the inclusion of the sex and gender variables in the content of the research. The study was STROBE compliant.

**Results:**

3,194 papers were published in 2011 and 6,781 in 2021, signed by at least one Spanish institution. The ratio of female authors per article was 0.39 in 2011 compared to 0.43 in 2021, reaching parity. Gender bias in the content of scientific articles, as measured by a 10-item checklist, showed an unfavorable score for perspective integration in both years (1.7/10 and 1.8/10, respectively). In terms of the Gender Balance Index in the analyzed samples of the papers (cells, animals or volunteers/patients), there was an increase from 29 % in 2011 to 43 % in 2021. Breast cancer research was dominated by female authors, whereas lung cancer research was predominantly by parity or male authorship, and no differences in author groups in colorectal cancer research were observed.

**Conclusions:**

Greater efforts and commitments are needed from government institutions, funding agencies, and academic structures to promote sex/gender integration in cancer research.

## Highlights

• Gender gap in cancer research improved with more female authors/articles last decade.
• Slightly Reduction in Gender Bias in cancer publications between 2021 and 2011.
• No gender perspective differences in research studies among female/male authorship.
• Breast cancer was studied by female majority and Lung cancer by parity or male majority.

## Introduction

Cancer is a leading cause of death worldwide, with an estimated 20 million new cases and 9.7 million deaths in 2022.^1^ Approximately 2.0 million people in the U.S. are expected to be diagnosed with cancer and 611,720 people will die of cancer in 2024.^2^ Generally, age-standardized cancer incidence and mortality rates are higher in men than in women, except for thyroid, gallbladder, and reproductive cancers, where women have higher rates.^3^ The history of oncology is a long and challenging one, marked by important advances in the understanding of cancer and the development of effective treatments. In this context, the promotion of high-quality research in oncology that includes the category of sex and/or gender is a priority.^4^^,^^5^

In the last decade, there has been an increase in United States and European investment in cancer research.^6^ Despite public and private policy efforts integrating gender perspective and professional equity in oncology over the last decade, effective integration remains a challenge, affecting both scientific career and research development. The women's percentage remains lower than men in terms of project coordination, principal investigators, and different professional levels of the research career.^7^ Consequently, it is still relevant to analyze the gender gap in cancer research and to verify whether the mandates of gender equality have been transferred to the scientific career and the professional health field.^8^ It is therefore essential to know the representation of the sexes in the authorship of published research since this is a true reflection of professional evolution, conditions access to leadership positions and allows us to evaluate the current situation in the field of oncology.

At the same time, it is necessary to guarantee that the advances in prevention, diagnosis, treatment, and prognosis resulting from cancer research are inclusive and representative, integrating the sex/gender dimension in each of the stages that make up the scientific method, ensuring the constitution of a diverse population sample that allows the analysis and extrapolation of the results generated for the entire community.^9^, ^10^, ^11^ This is particularly important given the new paradigm of cancer therapy, which relies on personalized and precise oncology.^12^^,^^13^

Based on previous antecedents, the authors hypothesized an increase in gender inclusion in oncology in the last decade. The objective of this work was to contrast the progress between 2011 and 2021 in cancer research, analyzing international publications signed by at least one Spanish institution to assess i) The gender gap in the research team; ii) The gender bias through the analysis of the scientific content and typology of cancer studied; and iii) The relationship between the presence of women in research teams and the implementation of the gender perspective in the development of cancer research.

## Material and methods

The reported study was appraised according to the STROBE guidelines for observational studies. The study units were scientific articles, and the study design consisted of the following key elements.

### Identification and selection of articles

The Web of Science (WoS) Core Collection ‒ Science Citation Index Expanded (SCIE) database was used for this study.

**Fig. 1:**
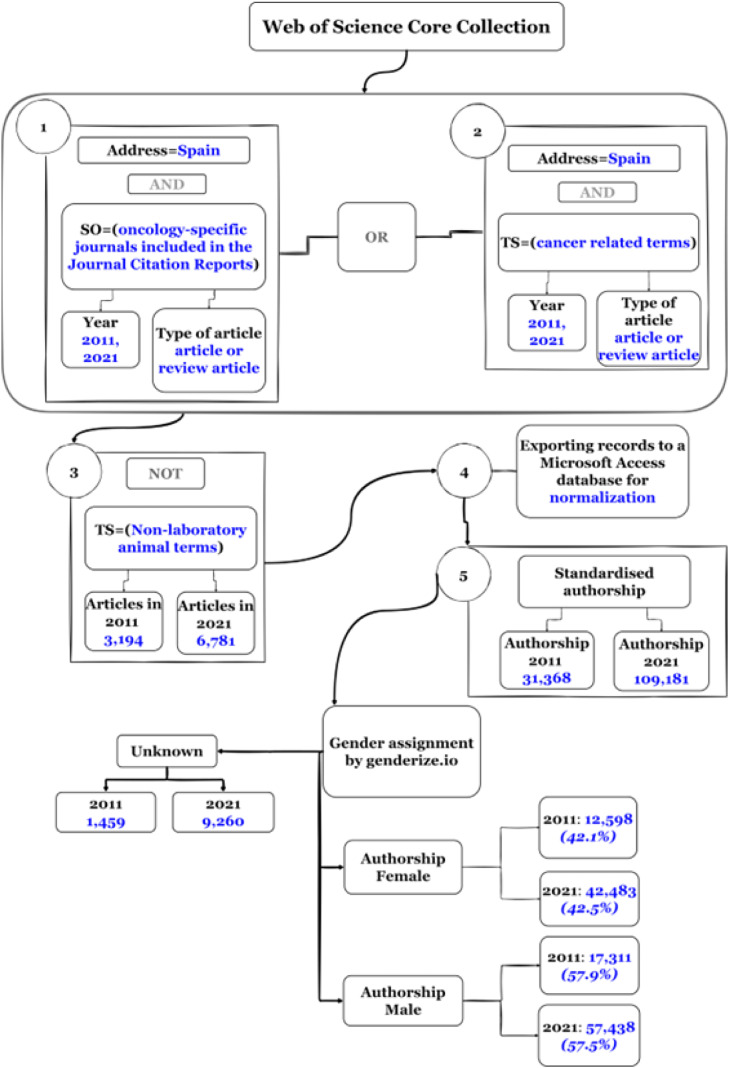
Flowchart of database creation to obtain the number of articles in 2011 and 2021, and authorship gender analysis process using the *genderize.io* method.

Articles and reviews on cancer were searched using an equation that included journals in the SCIE Oncology category, along with representative cancer terms and cancer typologies described by the U. S. National Cancer Institute (NCI)^14^ (Supplementary File 1). The search was limited to the years 2011 and 2021 and filtered by the term Spain in the address field to select documents signed by at least one Spanish institution. Using the proprietary SQL software “Bibliometricos”, the retrieved records were exported to a relational database in Microsoft Access. Subsequently, the authors' names were normalized manually (Fig. 1).

### Gender equity in the authorship of cancer publications with a Spanish signature

The statistical package Genderize.io^15^ was used to assign the sex of all authors. The Index of Female authors (IF) per paper was calculated by dividing the count of female authors by the total number of authors. Papers were then classified into gender Parity (P), when one gender constituted 40 % to 60 % of the total authors, or Female Majority (FM), and Male Majority (MM) authorship.

### Types of cancer studied

In an exploratory study, a random sample of 300 articles was obtained, 150 published in 2011 and 150 published in 2021. Papers were classified by cancer type based on title, abstract, and keywords, following the NCI criteria.^16^

### Inclusion of a gender perspective in scientific content

From all articles with identified authorship (Fig. 1), a random sample of 150 publications from 2011 (50 articles with majority female authorship; 50 articles with majority male authorship; 50 articles with parity authorship) and a similar sample from 2021, was carried out a comparative study of gender bias through the scientific content of the articles, classified according to the 3 groups of authorship. Inclusion criteria were articles with three or more authors and articles dealing with any type of cancer affecting both sexes, regardless of the degree of incidence in men or women. Exclusion criteria were articles dealing with cancers that physiologically affect only one sex, such as prostate, uterine, testicular, or ovarian cancer.

Subsequently, a checklist of 10 variables on the integration of the gender approach in the development of research was performed (Table 1). The 10 items that make up the questionnaire assess the integration of the sex/gender dimension throughout the research process and the preparation of the scientific article. Similarly, a document was drafted and agreed upon by the research team outlining the appropriate use of the criteria to be followed for their evaluation. The 300 articles were analyzed by two authors. In case of disagreement on the results of one of the examined items, the article was re-evaluated by the other authors until a consensus on the analysis was reached. Variables that were not reported in the article were coded as N/A (not applicable).

**Table 1:** Scores obtained in 2011 and 2021 on the variables that make up the Gender Inclusion Questionnaire to analyze the content of published oncology research.

Variables included in the questionnaire	Percentage of positive answer		p
	2011	2021	
V1. Are the title, keywords or abstract of the article inclusive of Sex/Gender?	25.3 %	16.7 %	0.066
V2. Has the Sex/Gender variable been evaluated in the research?	14 %	8.7 %	0.15
V3. Does the study design take into account the Sex/Gender variable?	30 %	33.3 %	0.54
V4. Did the authors provide detailed information on the estimated sample size tested?	2.7 %	9.3 %	0.015*
V5. Does the methodology guarantee that (possible) gender differences will be investigated? (data differentiated by Sex/Gender will be collected throughout the research cycle and will be part of the final publication)	8 %	8 %	0.73
V6. Are the groups involved in the project (e.g., samples, test groups) Gender-Balanced?	29.3 %	42.7 %	0.084
V7. Are the results shown taking into account the Sex/Gender variable?	34 %	30 %	0.46
V8. Have possible differentiated outcomes and impacts of research on women and men been considered?	10 %	8.7 %	0.75
V9. Have you reviewed the literature and other sources related to gender differences in the research field?	10 %	10.7 %	0.87
V10. If the research involves humans as research objects, has the relevance of gender to the research topic been analyzed?	6.7 %	4 %	0.28
Overall positive score	17 %	18 %	0.24

The Gender Balance Index (GBI) was obtained for the sample studied in each article (cohort of patients, healthy volunteers, experimental animals, etc.) according to the definition established by the European Commission,^8^ which defines gender balance as the presence of 40 % ‒60 % representation of both sexes and/or genders in the study. Therefore, the gender balance was considered to exist when the GBI was between 0.66 and 1.5, calculated as the number of females divided by the number of males in the sample analyzed (and similarly for female and male animals).

### Statistical analyses

Qualitative variables were described using frequencies and percentages, while quantitative variables were summarized using mean and standard deviation. The normality assumption was checked using the Shapiro-Wilks test and if normality was not confirmed, median and interquartile range were reported. For qualitative variables, the comparison of percentages between groups was studied using Fisher's exact test for dichotomous variables or the Chi-Square test for contingency tables with more than two categories.

To evaluate the inclusion of gender perspective in cancer research a global score was calculated by giving one point for each correctly answered question. This global score was studied using a linear model with year or authorship typology as independent factors. Individual variables were studied using generalized linear models from the binomial distribution with year and authorship typology as independent factors; p-values below 0.05 were considered significant. R software version 4.0.1 was used for all statistical analyses.

### Sample size

The margins of error with a 95 % Confidence Interval for the exploratory study vary between 8.98 % and 9.62 % in the analysis of the behavior of each authorship group in the decade. They vary between 13.30 % and 13.73 % when comparing the years 2011 and 2021.

## Results

### Gender equity among oncology researchers

A total of 3,194 papers were published in 2011 and 6,781 records in 2021, indicating a more than twofold increase in scientific production in cancer in the last decade (Fig. 1). The gender of the 140,549 authors of the publications obtained for both years was then assigned using the Genderize API. The total number of standardized authorships in 2011 was 31,368 signatories (12,598 females, 17,311 males, and 1,459 unknown) and in 2021 were retrieved 109,181 signatories (42,483 females, 57,438 males, and 9,260 unknown) (Fig. 1). No difference was observed between the two years in the female/male percentages, being around 40 % and 60 % the percentage of female and male signatories respectively. The IF signatories per article was 0.39 for 2011 compared to 2021, which increased to 0.43, reaching parity levels (0.4‒0.6).

**Fig. 2:**
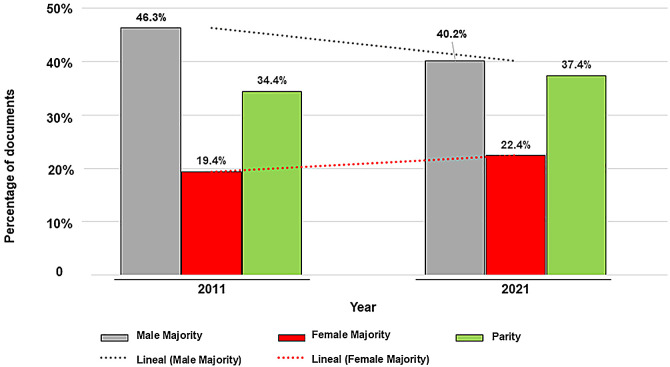
Distribution of the articles in the years 2011 and 2021 according to the three groups of authorship established among the authors of each article: Parity (P), mixed authorship with a Female Majority (FM), and mixed authorship with a Male Majority (MM).

The analysis of the evolution of publications categorized by authorship parity degree (Fig. 2) shows a trend towards an increase in 2021 vs. 2011 of publications signed by parity or female majority and a decrease in those signed by male majority.

### Gender differences in authorship of publications according to the type of cancer

**Fig. 3:**
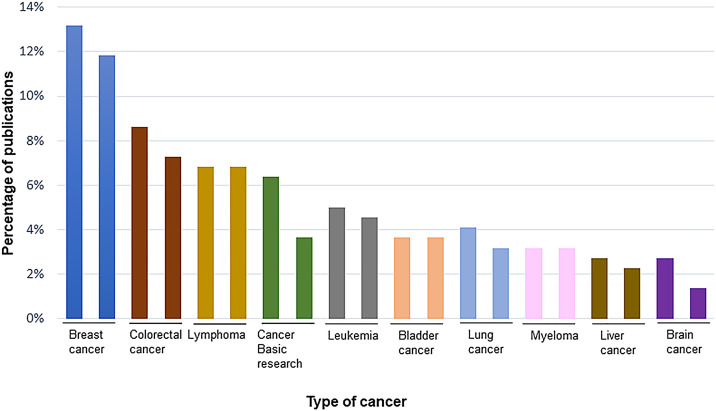
Analysis of the 10 most common cancers studied in oncology publications in 2011 and 2021.

The frequency of research on different types of cancer is shown in Fig. 3. Publications on breast cancer, colorectal cancer, lymphoma, and basic experiments dominate in both years.

**Fig. 4:**
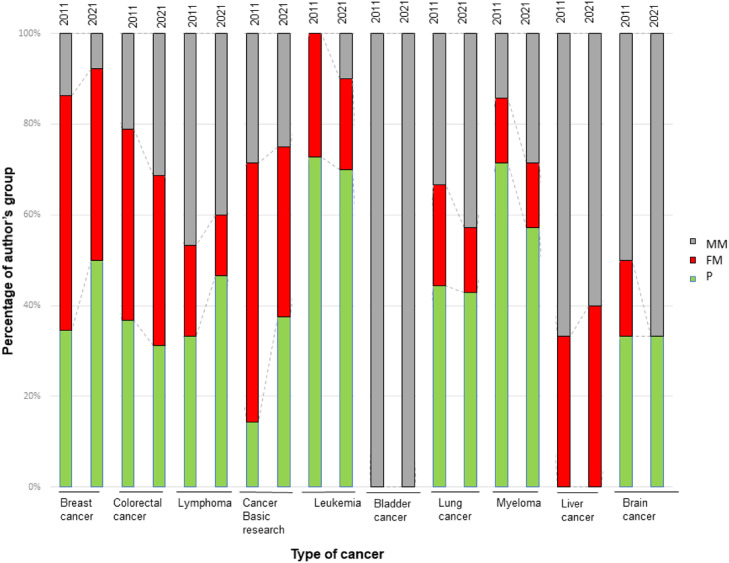
Analysis of the gender of their authors distributed in the three established authorship groups: Parity authorship (P), mixed authorship with a Male Majority (MM), and mixed authorship with a Female Majority (FM).

Fig. 4 shows the analysis of the different cancer types studied in the articles according to the three authorship groups. In breast cancer, the distribution of the paper's authorship is dominated by the FM- and P-groups, with this parity behavior becoming more pronounced in 2021 and the MM authorship decreasing (14 % in 2011, falling to 8 % in 2021). A different behavior is observed for hematological cancers (lymphoma, leukemia and myeloma) and lung cancer, where papers are mainly signed by P or MM authorship. In contrast, colorectal cancer research shows a similar distribution of the three authorship groups, with no differences between 2011 and 2021. Interestingly, in cancer basic research, the P-group was more than doubled in 2021 (38 % ) compared to 2011 (14 % ), while the FM group decreased. In contrast, MM authors dominate in publications on bladder cancer.

### Evolution of the gender perspective in cancer research content

For the analysis of the content of the articles, the average score per article has been calculated, bearing in mind that every “yes” in the variables of the questionnaire corresponds to one point, thus higher score indicates a stronger gender perspective in publications. The average global positive score for all articles in 2011 and 2021 was 1.7 points (0.17 SD) and 1.8 points (0.17 SD). For the overall analysis, the mean score between 2011 and 2021 was compared by Student's *t*-test, and the authorship group was compared by analysis of variance. All analyses were performed using R version 4.0.1. The cut-off point for significance was p < 0.05. The results of both analyses showed no differences in total scores between the two years (*t*-test, p = 0.24) and between authorship groups (ANOVA, p = 0.88).

**Fig. 5:**
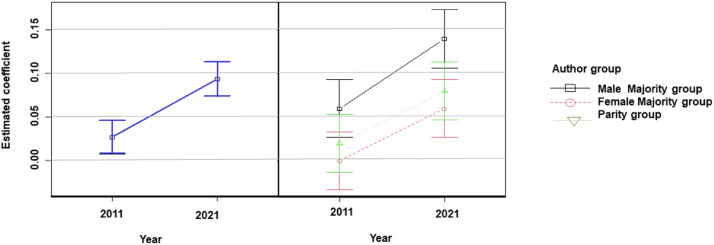
Gender bias in the content of oncology publications in 2011 and 2021. Analysis of variable 4.

In order to determine whether the year, or the group of authors, or both, conditioned the integration of the gender perspective in the research content, each variable of the questionnaire was analyzed separately. Only variables V3, V4, and V6 improved in 2021 compared to 2011 (Table 1). Significant differences were observed for variable 4, which refers to the design of published studies to check whether the sample size of the research is representative of the study population, according to the criteria of equity and inclusion of all genders. Fig. 5 shows that articles published in 2021 performed significantly better on this variable compared to 2011, regardless of the author group. For the other variables studied, no significant differences in compliance, and therefore in the scores obtained, were observed when comparing authorship groups or the interaction between years and authorship.

### Gender Balance Index in cancer research

An insight into the Gender Balance Index within the content of published articles was provided by the analysis of variable 6 (Table 1). The results obtained show that only 29 % of the papers in 2011 met the criteria for GBI, with a ratio between 0.6 and 1.5, compared to 43 % of the papers published in 2021.

## Discussion

The present study shows that scientific publications related to oncology increased over the last decade. In 2011, 3,194 papers on cancer were published and signed by at least one Spanish institution, being more than doubled by 2021 (6,781 publications) and indicating a growth in cancer scientific production throughout the decade. These data are consistent with the increase in the global scientific output on cancer obtained using the same search strategy, from 102,125 articles in 2011 to 206,860 articles in 2021.

The ratio of female authors per article was 0.39 in 2011 compared to 0.43 in 2021, reaching parity. Nevertheless, there is still a male majority in oncology authorship (57 % male vs. 43 % female), which changed little between 2011 and 2021. The present data are consistent with previous studies^17^, ^18^, ^19^ revealing gender gaps in health sciences^20^ and gender bias in research at professional and scientific levels,^21^ evidencing that discriminatory issues such as vertical (glass ceiling and scissors effect) and horizontal segregation (feminizing/masculinizing professional sectors) still persist in the scientific field.

These barriers have a negative impact on women's scientific careers in terms of project and research leadership^8^ and consequently on the number of publications, research areas, and publication impact.^18^ These gender gaps even affect the possibility of obtaining grants.^20^ The significant dropout of women in research careers is evident, with women compromising only 32.8 % of European researchers.^8^ Studies indicate women are underrepresented in science and receive less credit for their work across all science areas.^22^^,^^23^ Despite the progress made by women in scientific research in recent decades, it can be confirmed that gender inequality still persists in research teams,^17^ particularly in the field of oncology, where a persistent under-representation of women in scientific publications has also been observed. Publications with women's principal investigations remain scarce despite their increasing presence in the professional population in this field.^21^^,^^24^ The lower ratio of female to male oncologists is evidenced in ASCO's most recent 2022 State of Cancer Care in America report, where approximately one-third (35.8 % ) of oncologists are women, who also earn less than their male in their own racial or ethnic counterparts.^25^

Biomedical studies show also fewer female authors^26^ and, as in this study, the same upward trend in the number of female authors has been observed in the cardiovascular field in recent years.^27^

This means that by 2021, there will be slightly more equal or female majority treaties than in 2011. This trend may be the result of socio-political regulations aimed at the inclusion of women in science, innovation and health, including the Women's health research roadmap from the FDA Office of Women's Health (OWH),^28^ the NIH Strategic Plan for Women's Health Research,^29^ the Resolution of 2007 on equality between women and men in the European Union, and the Resolution of the European Parliament of 2008 on women and science, and the establishment of gender equality and gender mainstreaming in research as a priority to be achieved in the European Union by 2012.^8^ This last recommendation is envisaged in both the 2030 Agenda and the Sustainable Development Goals,^30^ as well as in the Strategy for Gender Equality 2020‒2025.^31^

In the present study, breast cancer, followed by colorectal cancer, lymphoma and lung cancer, are the most common cancer types examined, with a very similar pattern when comparing 2011 and 2021. These results are in line with the most commonly diagnosed cancers,^1^^,^^2^^,^^32^ with colorectal, breast, lung, prostate and bladder cancer being the most prominent. In men, the leading cancers are prostate, colorectal, lung and bladder cancer, while in women, the most common cancers diagnosed are breast and colorectal, followed by lung cancer.

Moreover, gender differences exist in survival 5-years after diagnosis being lower in men than in women (55 % and 62 % , respectively) for a set of 23 most common types of cancer.^33^ The presence of sex hormones in women, differences in the anatomical location of tumors, histological types and tumor aggressiveness, as well as differences in the prevalence of risk factors between the two sexes (tobacco and alcohol consumption), comorbidities and socio-cultural factors could contribute to these differences, urging research with data disaggregated by sex and gender.^33^^,^^34^

When analyzing the relationship between the type of cancer studied and the gender of the authors, breast cancer research is dominated by female authors, whereas lung cancer research is dominated by papers with an equal or male majority of authors. In contrast, there is equal participation in colorectal cancer research, the incidence of which is similar in men and women. Studies on bladder cancer are carried out by teams composed mainly of males, this, in turn, coincides with a higher incidence of this cancer in men.^3^ This effect seems to indicate a certain predilection in researchers for specific health topics they feel closer to,^35^ showing that between 60 % and 80 % of an individual's lived experiences and their emotional impact significantly influence the formation of their preferences. In this line, recent studies reveal that women are more concerned and knowledgeable about certain health issues, such as sexually transmitted diseases and sexually transmitted HPV infection, which are more popular because of their direct negative consequences for women, such as the development of cervical cancer, while most infected men remain asymptomatic.^36^

The gender bias analysis in the scientific content of the articles was carried out through a questionnaire, comparing their compliance between 2011 and 2021 and according to the 3 authorship groups. The average global score of the questionnaire indicates that there is still much improvement to be made, with only a 1.7 and 1.8 positive responses out of 10, in 2011 and 2021 respectively.

The increment in the number of articles complying with variable 4 (sample design) in 2021 stands out, as well as the increase in gender balance evaluated as variable 6 (42.7 % in 2021). The positive evolution of both items over the decade indicates an improvement in study design, allowing researchers and clinicians to extrapolate the results of research to their patients, taking into account their sex/gender. It also allows for a more detailed understanding of the population characteristics of the results of each trial, which can be applied with greater precision, moving towards personalized medicine and helping oncologists to make the right treatment decisions.^37^

The present findings suggest that cancer researchers, regardless of gender, do not often include a gender perspective. Although there has been some improvement in methodology, efforts to date are still inadequate. Therefore, it is crucial to prioritize training and awareness of gender perspective among cancer researchers, in order to advance all clinical aspects of the discipline (diagnosis, treatment, prognosis), prevention and epidemiology.

### Limitations

This study analyzed articles indexed in the WoS SCIE, so additional articles included in other databases may not have been retrieved.

The Genderize.io statistical package provides a probability of male or female gender based on an updated database that includes more than 200,000 names from over 79 countries and languages. While Genderize.io has been used in previous related work and provides a minimum accuracy of over 80 % , it has a limitation in that it cannot achieve 100 % accuracy.^38^

In the case that the evaluated papers showed an all-female or an all-male sample, it has been pointed out that there is no gender parity, and the GBI is equal to 0.

## Conclusions

The present study shows that authorship in the field of oncology is slightly reaching parity when comparing 2021 and 2011. In terms of the content of papers, there has been an increase in the Gender Balance Index in the samples analyzed. Nevertheless, this work highlights the persistence of gender gaps in research teams and gender bias in cancer research and stresses the need for urgent policy reforms to promote sex and gender training and reporting to improve the accuracy of research and its clinical application, with the aim of reducing inequalities among researchers and improving patient care.

## CRediT authorship contribution statement

**Rut Lucas-Domínguez:** Conceptualization, Methodology, Formal analysis, Investigation, Data curation, Writing – original draft, Writing – review & editing, Visualization, Supervision, Project administration, Funding acquisition. **Cristina Rius:** Conceptualization, Methodology, Software, Formal analysis, Investigation, Data curation, Writing – original draft, Writing – review & editing, Visualization. **Yiming Liu:** Methodology, Software, Investigation, Data curation, Writing – original draft, Writing – review & editing, Visualization. **Andrea Sixto-Costoya:** Conceptualization, Methodology, Software, Investigation, Data curation, Writing – original draft, Writing – review & editing, Visualization. **Juan Carlos Valderrama-Zurián:** Conceptualization, Formal analysis, Investigation, Writing – original draft, Writing – review & editing, Supervision, Project administration, Funding acquisition.

## Declaration of competing interest

The authors declare no conflicts of interest.

## Appendix Supplementary materials

Image, application 1
