# Supplementary material for: Sex and gender differences in cancer research and its application to clinical oncology and therapeutics
Source: Clinics (Sao Paulo). 2025 May 12;80:100670. doi: 10.1016/j.clinsp.2025.100670 (PMC12139217; doi:10.1016/j.clinsp.2025.100670)
Supplement: Supplementary file 1 [file mmc1.docx]

**CLINICS-D-24-00702_ Supplementary Material**

**Supplementary File 1** Search equation for publications in Oncology signed by at least one Spanish institution.

| Using the following search equation **((A OR B) NOT C) AND (Address: Spain).** |
| --- |
| The records were retrieved exclusively in the years 2011 and 2021. |
| The analysis equation was formulated as follows: |
| **Final search equation:** **((A OR B) NOT C) AND (Address: Spain)** |
| **Publication years: 2011 OR 2021** |
|  |
| Where **A** are the search terms representative of cancer along with the different typologies described by the NCI cancer types: |
| TS=(Adenocarcinoma OR Anticancer* OR Antimetastas* OR Antimetastatic* OR Antineoplas* OR Antioncolog* OR Antitumor* OR Antitumour* OR Astrocytom* OR Blastoma* OR Cancer* OR Carcinoid* OR Carcinom* OR Cholangiocarcinom* OR Chordom* OR Choriocarcinoma OR Craniopharyngiom* OR Ependymom* OR Esthesioneuroblastoma* OR “Gestational Trophoblastic Disease*” OR Glioblastoma OR Gliom* OR Histiocytom* OR (Langerhans NEAR/5 Histiocytosis) OR Leukaemia OR Leukem* OR Lymphom* OR Medulloblastoma OR Melanom* OR Mesotheliom* OR metastas* OR metastatic* OR “Mycosis Fungoides” OR “Myelodysplastic Syndrom*” OR Myelom* OR Neoplas* OR Neuroblastoma OR Oncogenic OR Oncolog* OR Oncoly* OR Osteosarcoma OR Papillomatosis OR Paragangliom* OR Pheochromocytom* OR Retinoblastoma OR Rhabdomyosarcoma OR Sarcom* OR “Sezary Syndrom*” OR Thymom* OR (Tumor* NEAR/5 malig*) OR (Tumour* NEAR/5 malig*) OR “Waldenström Macroglobulinemia” ) |
|  |
| Where **B** are the search terms that correspond to the journals in the Oncology category of the Science Citation Index Expanded from the JCR database. |
| SO=(“Acta Oncologica” OR “Advances in Cancer Research” OR “American Journal of Cancer Research” OR “American Journal of Clinical Oncology-Cancer Clinical Trials” OR “Annals of Oncology” OR “Annals of Surgical Oncology” OR “Annual Review of Cancer Biology-Series” OR “Anti-Cancer Agents in Medicinal Chemistry” OR “Anti-Cancer Drugs” OR “Anticancer Research” OR “Asian Pacific Journal of Cancer Prevention” OR “Asia-Pacific Journal of Clinical Oncology” OR “Biochimica Et Biophysica Acta-Reviews on Cancer” OR “Bladder Cancer” OR “Blood Cancer Journal” OR “Bmc Cancer” OR “Bone Marrow Niche Stem Cells and Leukemia Impact of Drugs Chemicals and The Environment*” OR “Brain Tumor Pathology” OR “Breast Cancer Research and Treatment” OR “Breast Cancer Research” OR “Breast Cancer” OR “Breast Cancer-Targets and Therapy” OR “Breast Care” OR “British Journal of Cancer” OR “Bulletin Du Cancer” OR “Ca-A Cancer Journal For Clinicians” OR “Cancer & Metabolism” OR “Cancer and Metastasis Reviews” OR “Cancer Biology & Medicine” OR “Cancer Biology & Therapy” OR “Cancer Biomarkers” OR “Cancer Biotherapy and Radiopharmaceuticals” OR “Cancer Causes & Control” OR “Cancer Cell International” OR “Cancer Cell” OR “Cancer Chemotherapy and Pharmacology” OR “Cancer Communications” OR “Cancer Control” OR “Cancer Cytopathology” OR “Cancer Discovery” OR “Cancer Epidemiology Biomarkers & Prevention” OR “Cancer Epidemiology” OR “Cancer Gene Therapy” OR “Cancer Genetics” OR “Cancer Genomics & Proteomics” OR “Cancer Imaging” OR “Cancer Immunology Immunotherapy” OR “Cancer Immunology Research” OR “Cancer Investigation” OR “Cancer Journal” OR “Cancer Letters” OR “Cancer Management and Research” OR “Cancer Medicine” OR “Cancer Microenvironment” OR “Cancer Nanotechnology” OR “Cancer Nursing” OR “Cancer Prevention Research” OR “Cancer Radiotherapie” OR “Cancer Research and Treatment” OR “Cancer Research” OR “Cancer Science” OR “Cancer Treatment Reviews” OR “Cancer” OR “Cancers” OR “Carcinogenesis” OR “Cellular Oncology” OR “Chinese Journal of Cancer Research” OR “Clinical & Experimental Metastasis” OR “Clinical & Translational Oncology” OR “Clinical and Translational Radiation Oncology” OR “Clinical Breast Cancer” OR “Clinical Cancer Research” OR “Clinical Colorectal Cancer” OR “Clinical Genitourinary Cancer” OR “Clinical Journal of Oncology Nursing” OR “Clinical Lung Cancer” OR “Clinical Lymphoma Myeloma & Leukemia” OR “Clinical Medicine Insights-Oncology” OR “Clinical Oncology” OR “Clinical Reviews in Oncogenesis” OR “Clinical Sarcoma Research” OR “Critical Reviews in Oncology Hematology” OR “Current Cancer Drug Targets” OR “Current Hematologic Malignancy Reports” OR “Current Oncology Reports” OR “Current Oncology” OR “Current Opinion in Oncology” OR “Current Problems in Cancer” OR “Current Treatment Options in Oncology” OR “Discover Oncology” OR “Ejc Supplements” OR “Ejso” OR “Endocrine-Related Cancer” OR “Esmo Open” OR “European Journal of Cancer Care” OR “European Journal of Cancer Prevention” OR “European Journal of Cancer” OR “European Journal of Gynaecological Oncology” OR “European Journal of Oncology Nursing” OR “European Journal of Oncology” OR “European Urology Oncology” OR “Experimental Hematology & Oncology” OR “Expert Review of Anticancer Therapy” OR “Familial Cancer” OR “Frontiers in Oncology” OR “Frontiers of Radiation Therapy and Oncology” OR “Future Oncology” OR “Gastric Cancer” OR “Genes Chromosomes & Cancer” OR “Gynecologic Oncology” OR “Head Neck Oncology” OR “Hematological Oncology” OR “Hematology-Oncology Clinics of North America” OR “Hereditary Cancer in Clinical Practice” OR “Hormones & Cancer” OR “Indian Journal of Cancer” OR “Infectious Agents and Cancer” OR “Integrative Cancer Therapies” OR “International Journal of Cancer” OR “International Journal of Clinical Oncology” OR “International Journal of Gynecological Cancer” OR “International Journal of Oncology” OR “International Journal of Radiation Oncology Biology Physics” OR “Jacc: Cardiooncology” OR “Jama Oncology” OR “Japanese Journal of Clinical Oncology” OR “Jco Oncology Practice” OR “Jco Precision Oncology” OR “Jnci-Journal of The National Cancer Institute” OR “Journal For Immunotherapy of Cancer” OR “Journal of Adolescent and Young Adult Oncology” OR “Journal of Bone Oncology” OR “Journal of Breast Cancer” OR “Journal of Buon” OR “Journal of Cancer Education” OR “Journal of Cancer Research and Clinical Oncology” OR “Journal of Cancer Research and Therapeutics” OR “Journal of Cancer Survivorship Research and Practice” OR “Journal of Cancer Survivorship” OR “Journal of Cancer” OR “Journal of Clinical Oncology” OR “Journal of Environmental Science and Health Part C Environmental Carcinogenesis Ecotoxicology Reviews” OR “Journal of Environmental Science and Health Part C-Toxicology and Carcinogenesis” OR “Journal of Experimental & Clinical Cancer Research” OR “Journal of Gastric Cancer” OR “Journal of Gastrointestinal Oncology” OR “Journal of Geriatric Oncology” OR “Journal of Gynecologic Oncology” OR “Journal of Hematology & Oncology” OR “Journal of Hepatocellular Carcinoma” OR “Journal of Mammary Gland Biology and Neoplasia” OR “Journal of Neuro-Oncology” OR “Journal of Oncology Pharmacy Practice” OR “Journal of Oncology Practice” OR “Journal of Oncology” OR “Journal of Pediatric Hematology Oncology” OR “Journal of Pediatric Oncology Nursing” OR “Journal of Surgical Oncology” OR “Journal of The National Cancer Institute” OR “Journal of The National Comprehensive Cancer Network” OR “Journal of Thoracic Oncology” OR “Lancet Oncology” OR “Leukemia & Lymphoma” OR “Leukemia Research” OR “Leukemia” OR “Liver Cancer” OR “Lung Cancer” OR “Medical Oncology” OR “Melanoma Research” OR “Molecular Cancer Research” OR “Molecular Cancer Therapeutics” OR “Molecular Cancer” OR “Molecular Carcinogenesis” OR “Molecular Oncology” OR “Molecular Therapy-Oncolytics” OR “Nature Cancer” OR “Nature Reviews Cancer” OR “Nature Reviews Clinical Oncology” OR “Neoplasia” OR “Neoplasma” OR “Neuro-Oncology” OR “Npj Breast Cancer” OR “Npj Precision Oncology” OR “Nutrition and Cancer-An International Journal” OR “Oncogene” OR “Oncogenesis” OR “Oncoimmunology” OR “Oncologie” OR “Oncologist” OR “Oncology Biology Physics” OR “Oncology Letters” OR “Oncology Nursing Forum” OR “Oncology Reports” OR “Oncology Research and Treatment” OR “Oncology Research” OR “Oncology” OR “Oncology-New York” OR “Oncotarget” OR “Oncotargets and Therapy” OR “Onkologe” OR “Onkologie” OR “Oral Oncology” OR “Pathology & Oncology Research” OR “Pediatric Blood & Cancer” OR “Pediatric Hematology and Oncology” OR “Pigment Cell & Melanoma Research” OR “Practical Radiation Oncology” OR “Prostate Cancer and Prostatic Diseases” OR “Psycho-Oncologie” OR “Psycho-Oncology” OR “Radiation Oncology” OR “Radiology and Oncology” OR “Radiotherapy and Oncology” OR “Recent Patents on Anti-Cancer Drug Discovery” OR “Seminars in Cancer Biology” OR “Seminars in Oncology Nursing” OR “Seminars in Oncology” OR “Seminars in Radiation Oncology” OR “Strahlentherapie Und Onkologie” OR “Supportive Care in Cancer” OR “Surgical Oncology Clinics of North America” OR “Surgical Oncology-Oxford” OR “Targeted Oncology” OR “Technology in Cancer Research & Treatment” OR “Therapeutic Advances in Medical Oncology” OR “Thoracic Cancer” OR “Translational Cancer Research” OR “Translational Lung Cancer Research” OR “Translational Oncology” OR “Trends in Cancer” OR “Tumori Journal” OR “Uhod-Uluslararasi Hematoloji-Onkoloji Dergisi” OR “Urologic Oncology-Seminars and Original Investigations” OR “World Journal of Gastrointestinal Oncology” OR “World Journal of Surgical Oncology” OR “Wspolczesna Onkologia Contemporary Oncology”) |
|  |
| Where **C** are the search terms that correspond to animals not used in laboratory research. |
| TS=(bird* OR bovine* OR canine* OR cat* OR dog* OR dolphin* OR feline* OR goat* OR horse* OR ovine OR pig* OR porcine OR shark* OR sheep OR turtle*) OR TI=( veterinar*) OR SO=(animal* OR avian* OR bovine* OR parasite* OR porcine* OR ruminant* OR veterinar* OR wildlife) |
